# Targeting miR-21 to Overcome P-glycoprotein Drug Efflux in Doxorubicin-Resistant 4T1 Breast Cancer

**DOI:** 10.34133/bmr.0095

**Published:** 2024-10-21

**Authors:** Eun Hye Kim, Youngri Ryu, Jiwoong Choi, Daeho Park, Jong Won Lee, Sung-Gil Chi, Sun Hwa Kim, Yoosoo Yang

**Affiliations:** ^1^ Medicinal Materials Research Center, Biomedical Research Division, Korea Institute of Science and Technology (KIST), Seoul 02792, Republic of Korea.; ^2^ Department of Life Sciences, Korea University, Seoul 02841, Republic of Korea.; ^3^ KU-KIST Graduate School of Converging Science and Technology, Korea University, Seoul 02841, Republic of Korea.; ^4^ Division of Bio-Medical Science and Technology, KIST School, University of Science and Technology, Seoul 02792, Republic of Korea.

## Abstract

Acquired resistance to chemotherapy is a major challenge in the treatment of triple-negative breast cancer (TNBC). Despite accumulated evidence showing microRNA-21 (miR-21) as a vital regulator of tumor progression, the role of miR-21 in modulating the multidrug resistance of TNBC remains obscure. In this study, we demonstrate that miR-21 affects chemoresistance in 4T1 TNBC cells in response to doxorubicin (DOX) by regulating the P-glycoprotein (P-gp) drug efflux pump. Overexpression of miR-21 in the 4T1 cells markedly reduced their sensitivity to DOX, impeding DOX-promoted cell death. We employed anti-miR-21 oligonucleotide conjugated with a PD-L1-binding peptide (P21) for targeted delivery to 4T1 tumor cells. The selective down-regulation of miR-21 in 4T1 TNBC led to the reversal of P-gp-mediated DOX resistance by up-regulating phosphatase and tensin homolog (PTEN). Our study highlights that miR-21 is a key regulator of drug efflux pumps in TNBC, and targeting miR-21 could enhance DOX sensitivity, offering a potential therapeutic option for patients with DOX-resistant TNBC.

## Introduction

Triple-negative breast cancer (TNBC) represents a highly heterogeneous and aggressively metastatic disease, accounting for approximately 15 to 20% of all diagnosed breast cancer cases worldwide [1,2]. Characterized by the absence of estrogen receptor, progesterone receptor, and human epidermal growth factor receptor 2 expression, targeted therapies for TNBC are limited, resulting in a poor prognosis [3]. Consequently, the current standard first-line treatment for TNBC patients primarily relies on chemotherapy drugs such as doxorubicin (DOX) [4]. However, prolonged exposure to DOX can paradoxically induce acquired drug resistance in cells through adaptive cellular responses, presenting a significant clinical challenge in TNBC treatment [5].

The development of multidrug resistance (MDR) to chemotherapy serves as a critical impediment to achieving successful outcomes in cancer therapy [6]. Various mechanisms contribute to MDR, including cell cycle alterations, up-regulation of drug transporters, alterations in drug metabolism, and enhanced DNA repair processes [7]. Among these mechanisms, we particularly focused on the overexpression of adenosine triphosphate (ATP)-dependent P-glycoprotein (P-gp) drug efflux pump, encoded by the MDR1 gene [8,9]. P-gp overexpression diminishes intracellular drug accumulation, thereby reducing chemotherapy efficacy and contributing to treatment resistance [10,11]. Despite the potential of exogenous P-gp inhibitors, their clinical use is hindered by nonspecific tissue-targeting and associated side effects [12]. First- and second-generation P-gp inhibitors, including verapamil, cyclosporine, and valspodar, have faced challenges in clinical trials due to adverse effects on normal cells and the need for high doses to effectively block P-gp activity [13–15]. Even with advancements such as third-generation inhibitors like tariquidar, unresolved safety concerns persist, leading to unsatisfactory clinical outcomes [16]. Thus, exploring endogenous molecules capable of effectively reversing MDR by regulating P-gp expression holds significant importance.

MicroRNAs (miRNAs), a class of endogenous small noncoding RNAs, play crucial roles in regulating gene expression across various cellular processes and molecular pathways [17,18]. Recent studies have highlighted that miRNAs are key endogenous negative modulators of drug resistance by regulating drug efflux and apoptosis [19,20]. In particular, aberrant expression levels of microRNA-21 (miR-21), which are highly expressed in most cancer cells, have been closely associated with cancer progression and DOX resistance [21]. Down-regulation of miR-21 has been shown to restore the sensitivity of TNBC cells to DOX by modulating the phosphatase and tensin homolog (PTEN) expression [22,23]. Recent studies have emphasized that reducing miR-21 expression in TNBC patients is critical for improving survival rates, enhancing treatment efficacy, and overcoming drug resistance [24–26]. This underscores the potential of miR-21-targeted strategies to improve treatment outcomes in TNBC by mitigating drug resistance. While existing research has primarily focused on the relationship between miR-21 and PTEN, there has been insufficient exploration into how PTEN mediates the reversal of drug resistance. Our research has identified a key mechanism by which reactivating PTEN through miR-21 inhibition can alleviate drug resistance. By targeting miR-21 to inhibit its expression, PTEN levels can be reactivated, leading to the regulation of the downstream P-gp drug efflux pump. Based on prior research on miR-21’s role in DOX resistance, this mechanism offers a more effective strategy for treating various TNBC models [27–29] and provides significant insights into overcoming drug resistance.

In this study, we employed a previously developed peptide–oligonucleotide conjugate, combining a PD-L1-binding peptide with anti-miR-21 (P21), as a therapeutic approach for tumor-targeted delivery of anti-miR-21 in a DOX-resistant 4T1 TNBC model [30]. Our findings elucidated the intricate role of miR-21 in DOX resistance and demonstrated that P21 enhances DOX sensitivity by deactivating the P-gp efflux pump, resulting in a potent anticancer effect. Consequently, these results suggest that miR-21 expression levels serve as predictive biomarkers for DOX resistance and potential therapeutic targets in DOX-resistant TNBC models.

## Materials and Methods

### Cell culture

TNBC mouse cell line 4T1 was obtained from the American Type Culture Collection (USA) and cultured in Roswell Park Memorial Institute (RPMI)-1640 (Welgene, Republic of Korea) supplemented with 10% fetal bovine serum (FBS; Gibco, USA) and 1% antibiotic–antimycotic (Gibco, USA). All cell lines were incubated at 37 °C in a humidified atmosphere with 5% CO_2_.

To establish a DOX-resistant 4T1 cell line (4T1/R), 4T1 cells were continuously treated with gradually increasing concentrations of DOX over 1 year. When the cells reached an appropriate confluence at a certain concentration, the dose of DOX was increased by 10 nM up to a final concentration of 500 nM. After 5 sequential treatments with a final concentration of 500 nM DOX, stable 4T1/R cells resistant to DOX were obtained. 4T1/R cells were maintained in RPMI-1640 supplemented with 10% FBS (Gibco, USA) and 1% antibiotic–antimycotic (Gibco, USA).

### Cell viability assay

To investigate the cell viability, 5 × 10^3^ 4T1 and 4T1/R cells were seeded in 96-well plates and incubated for 24 h at 37 °C. After stabilization, the cells were treated with different concentrations of DOX (ranging from 0.001 to 50 μM) for 24 h. Following treatment, 10 μl of 3-(4,5-dimethylthiazol-2-yl)−2,5-diphenyltetrazolium bromide (MTT) solution (Sigma-Aldrich, USA) was added to each well at a final concentration of 0.5 mg/ml and the cells were incubated for 4 h. Then, 100 μl of solubilization buffer was added to each well, and the plates were incubated overnight. Finally, the cell viability was measured using a microplate reader (SpectraMax 34, Molecular Devices, USA) at a wavelength of 570 nm.

### Colony formation assay

To assess colony-forming ability, 5 × 10^3^ 4T1 and 4T1/R cells were seeded in a 6-well plate for overnight at 37 °C. Following treatment with DOX for 24 h, the medium was replaced with fresh medium, and the cells were cultured for an additional 8 d. The colonies formed were then fixed using 4% paraformaldehyde for 15 min at room temperature, stained with 0.5% crystal violet for 15 min, and subsequently counted to quantify colony formation.

### Flow cytometry

For flow cytometry analysis, 2 × 10^5^ 4T1 and 4T1/R cells were seeded into a 35-mm glass-bottom dish and incubated overnight at 37 °C. The cells were then harvested and preblocked with Fc blocker (BD Pharmingen, USA, clone 2.4G2, #553142) for 15 min at 4 °C. To quantify the expression levels of PD-L1 on the cell surface, single-cell suspensions were incubated with allophycocyanin-conjugated PD-L1 antibodies (clone 10F.9G2, #124312) for 30 min at 4 °C. After incubation, the cells were washed 3 times with phosphate-buffered saline (PBS) to remove unbound antibodies. Finally, the stained cells were resuspended in PBS for flow cytometry analysis. All samples were analyzed by a CytoFlex flow cytometer (Beckman Coulter, USA) using FlowJo (v10) software.

### Quantitative reverse transcription polymerase chain reaction

To examine the relative expression level of miR-21, 2 × 10^5^ 4T1 and 4T1/R cells were seeded into a 35-mm glass-bottom dish and incubated overnight. Total RNA was isolated from 4T1 and 4T1/R cells using miRNeasy mini kit (Qiagen, Germany) according to the manufacturer’s protocol. RNA concentration and purity were quantified using a NanoDrop spectrophotometer (Thermo Fisher Scientific, USA). Subsequently, the complementary DNAs were synthesized from the above RNAs using the Mir-X miRNA First-Strand Synthesis (Takara Bio, Japan). Finally, quantitative reverse transcription polymerase chain reaction (qRT-PCR) was performed using the StepOnePlus Real-Time PCR system with TOPreal SYBR Green qPCR kits (Enzynomic, Republic of Korea). The relative expression of miR-21 was normalized with U6 RNA and evaluated using 2^−∆∆Ct^ method. All qRT-PCR protocols for miRNA are performed following the manufacturer’s instructions. All primers used in this experiment are listed in Table.

**Table.** qRT-PCR primer sequences

Gene	Sequence (5′-3′)
*miR-21*	F: 5′-CTCGCTTCGGCAGCACA-3′ R: 5′-GTGCAGGGTCCGAGGT-3′
*U6*	F: 5′-AGACTAGCTTATCAGACTGA-3′ R: 5′-GTGCAGGGTCCGAGGT-3′
*PTEN*	F: 5′-TCATTACACCAGTCCGTCCCT-3′ R: 5′-TGAAGACCATAACCCACCACA-3′
*MDR-1*	F: 5′-GCTGGTTTGATGTGCACGATGTTGG-3′ R: 5′-ATTTTGTCACCAATTCCTTCATTAA-3′
*18s*	F: 5′-TTCCGATAACGAACGAGACTCT-3′ R: 5′-TGGCTGAACGCCACTTGTC-3′

F, forward; R, reverse

### Cellular uptake

To assess the cellular uptake level of DOX, 2 × 10^5^ 4T1 and 4T1/R cells were plated in 35-mm glass-bottom dishes. After stabilization, the cells were exposed to 2 μM DOX in a serum-free medium for 5 h. Subsequently, the cells were washed with PBS to remove the nonspecific binding, fixed with 4% formaldehyde for 10 min, and stained with Hoechst 33342 (Invitrogen, USA) for 10 min at room temperature. For fluorescence imaging, the cells were visualized using a CS SP8 confocal microscope (Leica TCS SP5; Leica, Germany). To quantify the cellular uptake of P21 and DOX, 4T1/R cells were treated with 150 nM P21 and 2 μM DOX in serum-free media for 6 and 8 h, respectively. The visualization procedure was performed identically to the previously described method.

For flow cytometry analysis of cellular uptake, 3 × 10^5^ 4T1 and 4T1/R cells were seeded into 35-mm glass-bottom dishes and incubated overnight. Afterward, the cells were collected and treated with 150 nM P21 and 2 μM DOX in serum-free media for 1 h. Subsequently, all samples were analyzed using a CytoFlex flow cytometer (Beckman Coulter, USA) using FlowJo (v10) software.

### Western blotting

For Western blot analysis of cells, 1 × 10^5^ 4T1 and 4T1/R cells were seeded into 6-well plates. After incubation, the cells were lysed using radioimmunoprecipitation assay buffer (Thermo Fisher Scientific, USA) supplemented with a 1% protease/phosphatase inhibitor cocktail, and maintained on ice for 20 min. The lysates were then centrifuged at 12,000*g* for 20 min to remove insoluble materials. After the protein concentration was quantified using the BCA Protein Assay kit (Bio-Rad, USA), 20 μg of total protein lysate was electrophoresed in 10% sodium dodecyl sulfate–polyacrylamide gel electrophoresis (SDS-PAGE) gel and transferred to nitrocellulose membrane blots. The membranes were blocked with 5% skim milk blocking buffer, incubated with primary antibodies overnight at 4 °C, and then exposed to secondary antibodies for 1 h at room temperature. After that, protein signals were visualized using enhanced chemiluminescence substrate (Bio-Rad, USA) and detected with chemiluminescence (iBright; Invitrogen, USA). The antibodies used in this study were as follows: PTEN (Cell Signaling Technology, USA, #9959; dilution 1:1,000), P-gp (Abcam, USA, #170904; dilution 1:1,000), glyceraldehyde-3-phosphate dehydrogenase (GAPDH) (GeneTex, USA, TX100118; 1:1,000), anti-rabbit immunoglobulin G (IgG)-horseradish peroxidase (HRP) antibody (GeneTex, USA, GTX213110–01; 1:2,000), and anti-mouse IgG-HRP antibody (GeneTex, USA, GTX213111 01; 1:2,000).

### Apoptosis assay

To validate the cellular apoptosis/necrosis induced by P21, apoptosis was measured using an Annexin V/propidium iodide (PI) staining assay kit (Abcam, USA, #ab14085). 4T1 or 4T1/R cells (1.5 × 10^5^) were seeded into a 35-mm glass-bottom dish and incubated overnight at 37 °C. Next, 4T1 or 4T1/R cells were treated with 0.5 μM DOX, 150 nM P21, or P21 + DOX for 24 or 48 h. The cells were harvested and resuspended with 500 μl of binding buffer. Subsequently, they were incubated with fluorescein isothiocyanate-conjugated Annexin V and PI for 5 min at room temperature in the dark. Following staining, the cells were analyzed for apoptosis and necrosis using a CytoFlex flow cytometer (Beckman Coulter, USA). Data acquisition and analysis were performed using FlowJo (v10) software, allowing for the quantification of the proportion of early apoptotic (Annexin V positive, PI negative), late apoptotic (Annexin V positive, PI positive), and necrotic (Annexin V negative, PI positive) cells within the sample.

### Animal experiments

Mice were purchased from the Orient Bio (Republic of Korea). All animal experimental procedures were performed in a specific pathogen-free animal facility at the Korea Institute of Science and Technology (KIST), in strict accordance with the relevant laws and institutional guidelines of the Institutional Animal Care and Use Committee (IACUC) of KIST (approval number: KIST-2022-04-072).

Six-week-old male BALB/c nude mice were subcutaneously inoculated with 2 × 10^6^ 4T1 or 4T1/R cells into the left flank. After 13 d of tumor inoculation, 4T1- or 4T1/R-bearing mice were sacrificed, and single cells were isolated from tumor tissues using a Tumor Dissociation Kit (Miltenyi Biotec, USA) for analyzing the mRNA expression levels of miR-21, PTEN, and P-gp. Furthermore, to evaluate the tumor regression efficacy of DOX in both tumor-bearing models, 2 × 10^6^ 4T1 or 4T1/R cells were subcutaneously inoculated into the left flanks of nude mice. When the tumor volumes were approximately 70 to 90 mm^3^, the mice were intratumorally administered with 1 mg/kg DOX. The injection was repeated 3 more times at 3-d intervals.

For combination therapy with P21 and DOX, 6-week-old male BALB/c nude mice were subcutaneously inoculated with 2 × 10^6^ 4T1/R cells into the left flank. Following 8 d of tumor growth to achieve an approximate volume of 50 to 60 mm^3^, the mice were randomly assigned to 1 of 3 treatment groups: control (Con), P21, and DOX in combination with P21. Mice were treated with intravenous injections of 0.78 mg/kg (2 nmol of anti-miR-21) P21 3 times at 3-d intervals on days 8, 11, and 14 and intratumorally injected with 1 mg/kg DOX 3 times at 3-d intervals on days 9, 12, and 15. Tumor growths and body weights were monitored every 2 d. Tumor volume (mm^3^) was calculated with calipers using the following formula: (width^2^ × length)/2.

### Histological analysis

For immunofluorescence staining of Ki67, tumor tissues obtained from the 4T1/R model were extracted the day after the last injection. The excised tumor tissues were fixed in a 4% paraformaldehyde solution, and the dehydrated tissues were embedded in paraffin and cut into 8-μm-thick sections using a rotary microtome (Accu-cut SRM200 Rotary Microtome, Japan). Tumor tissue slides were rehydrated with antigen retrieval step using 1× citrate solution (Abcam, UK) and then blocked with a 1% bovine serum albumin for 15 min at room temperature. The slides were then washed 3 times with PBS and incubated with phycoerythrin fluorescent-conjugated anti-Ki67 (clone 16A8, #652403) at 4 °C overnight. Nuclei were stained with Hoechst 33342 (Invitrogen, USA) for 10 min at room temperature. All images were analyzed using a CS SP8 confocal microscope (Leica TCS SP5; Leica, Germany).

For the detection of apoptotic cells in mouse tumor tissues, the sections were stained with terminal deoxynucleotidyl transferase-mediated deoxyuridine triphosphate nick end labeling (TUNEL) assay (Promega, USA, G3250) following the manufacturer’s instructions. All data were analyzed to quantify the relative fluorescence intensity compared to controls.

### Statistical analysis

All statistical analyses were performed using Prism 8 (GraphPad) and presented as the mean ± standard deviation (SD). Statistical significance between groups was calculated using Student’s *t* tests or 1-way (or 2-way) analysis of variance (ANOVA) followed by Tukey’s multiple comparisons test (*P < 0.05, **P < 0.01, ***P < 0.001, ****P < 0.0001).

## Results

### miR-21 confers resistance to DOX in 4T1 cells through modulation of P-gp expression

**Fig. 1.**
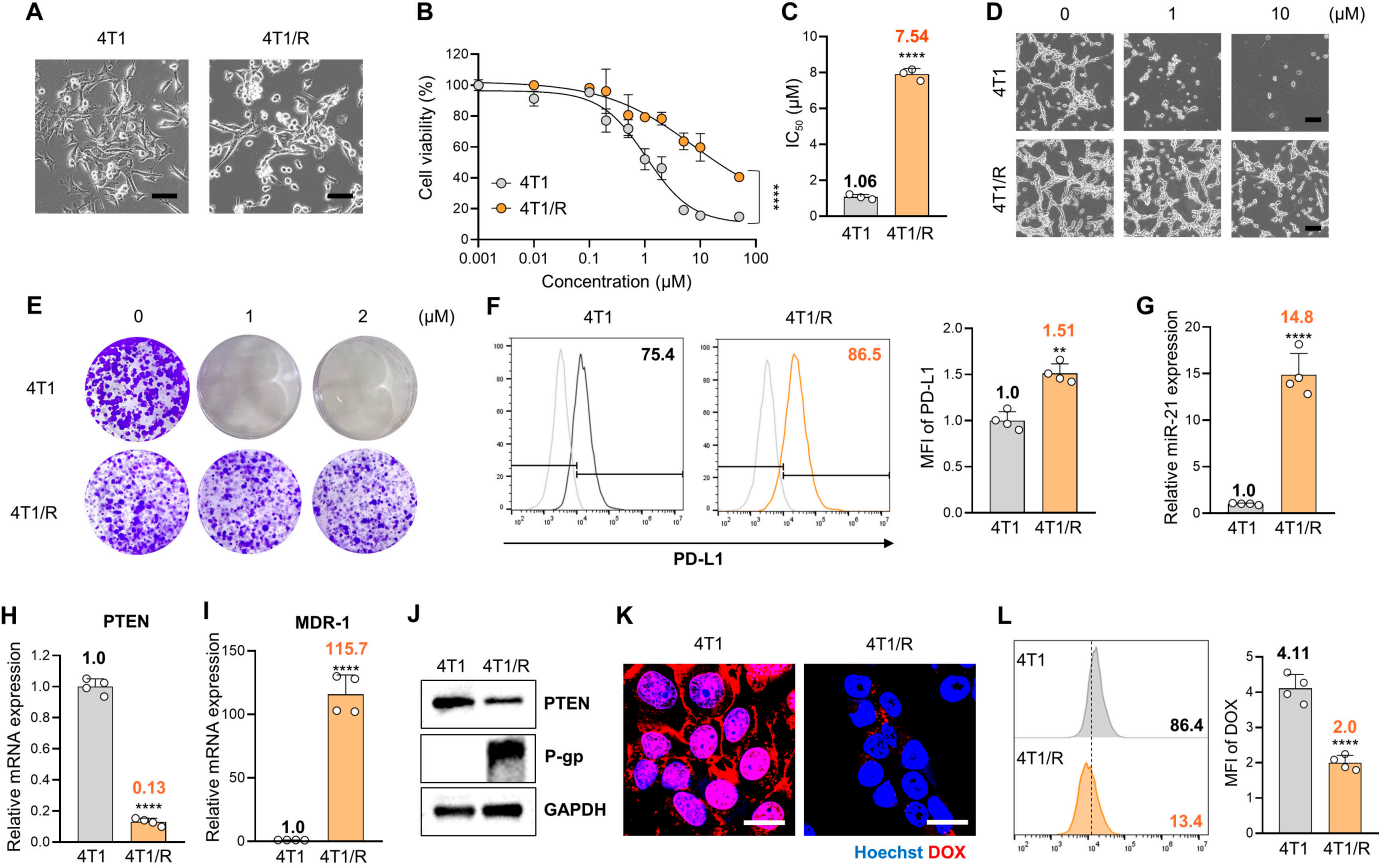
Effect of miR-21 on the regulation of P-gp expression in DOX-resistant 4T1 cells. (A) Morphological differences between 4T1 and 4T1/R cells. (B) Viability of 4T1 and 4T1/R cells following treatment with gradient doses of DOX (0 to 100 μM) for 24 h (*n* = 3/group). (C) The IC_50_ values of 4T1 and 4T1/R cells to DOX according to the viability curves were indicated (*n* = 3/group). (D) Representative cell morphology in 4T1 and 4T1/R cells after treatment with the same DOX concentration (*n* = 3/group). (E) Colony formation analysis of 4T1 and 4T1/R cells after treatment with the same DOX concentration (*n* = 3/group). (F) Expression of PD-L1 in 4T1 and 4T1/R cells measured by flow cytometry (gray: isotype control). Data are presented as the relative mean fluorescent intensity (MFI) values (*n* = 3/group). (G) Relative miR-21 expression levels measured by qRT-PCR in 4T1 and 4T1/R cells. All samples were normalized to U6 expression (*n* = 4/group). (H and I) Relative PTEN and MDR-1 mRNA expression levels in 4T1 and 4T1/R cells. All samples were normalized according to 18s RNA expression (*n* = 4/group). (J) Western blot analysis of PTEN and P-gp abundance in 4T1 and 4T1/R cells (*n* = 3/group). (K) Representative confocal images of uptake by 4T1 and 4T1/R cells after treatment with DOX (2 μM) for 4 h. The nuclei were stained with Hoechst 33342 (blue) (scale bar, 50 μm; *n* = 3/group). (L) Representative flow cytometric histogram of intracellular uptake of DOX in 4T1 and 4T1/R cells after treatment with DOX (2 μM) for 1 h. Data are presented as the relative MFI values (*n* = 3/group).

To establish DOX-resistant 4T1 breast cancer cell lines, murine 4T1 cells were subjected to prolonged exposure to increasing concentrations of DOX. After 5 subsequent treatments with a final DOX concentration of 500 nM, the DOX-resistant 4T1 cells (4T1/R) were successfully developed, displaying markedly different cell morphology compared to the 4T1 parental cell lines (Fig. 1A). The IC_50_ (median inhibitory concentration) values of DOX for 4T1 and 4T1/R cells were measured at 1.06 and 7.54 μM, respectively, indicating that the 4T1/R cell line acquired 7.11-fold resistance to DOX compared to the parental cell line (Fig. 1B and C). Subsequently, both cell lines were treated with various concentrations of DOX and observed morphological changes indicative of cell sensitivity to the drug. As a result, 4T1/R cells exhibited greater resistance to DOX than 4T1 cells, as lower concentrations of DOX did not significantly affect the viability of 4T1/R cells (Fig. 1D). Moreover, 4T1/R cells demonstrated a higher colony-forming ability compared to 4T1 cells when exposed to the same concentration of DOX (Fig. 1E). In line with recent studies demonstrating increased PD-L1 expression induction in 4T1 cells following treatment with anticancer chemotherapeutic agents [31], we observed a slight enhancement in PD-L1 expression in 4T1/R cells compared to 4T1 cells (Fig. 1F).

Furthermore, we investigated the relationship between DOX resistance and miR-21-5p (miR-21) expression. Our results showed a notable up-regulation of miR-21 expression in 4T1/R cells compared to 4T1 cells, indicating a correlation between miR-21 overexpression and DOX resistance of 4T1 cells (Fig. 1G). Additionally, we observed reduced PTEN expression and up-regulated P-gp levels, both associated with miR-21 signaling, in 4T1/R cells compared to 4T1 cells at both the mRNA and protein levels (Fig. 1H to J). The increased expression of P-gp proteins, which control drug efflux, resulted in decreased intracellular accumulation of DOX in the nucleus of 4T1/R cells, with minimal presence in the cytoplasm (Fig. 1K). Flow cytometry analysis also revealed impaired cellular uptake of DOX in DOX-resistant cells compared to the parental cells (Fig. 1L). Collectively, the elevated expression of P-gp in DOX-resistant cells contributed to reduced intracellular DOX accumulation by impairing its intracellular uptake.

### Inhibition of miR-21 in DOX-resistant 4T1 cells improves sensitivity to DOX by deactivating the P-gp efflux pump

**Fig. 2.**
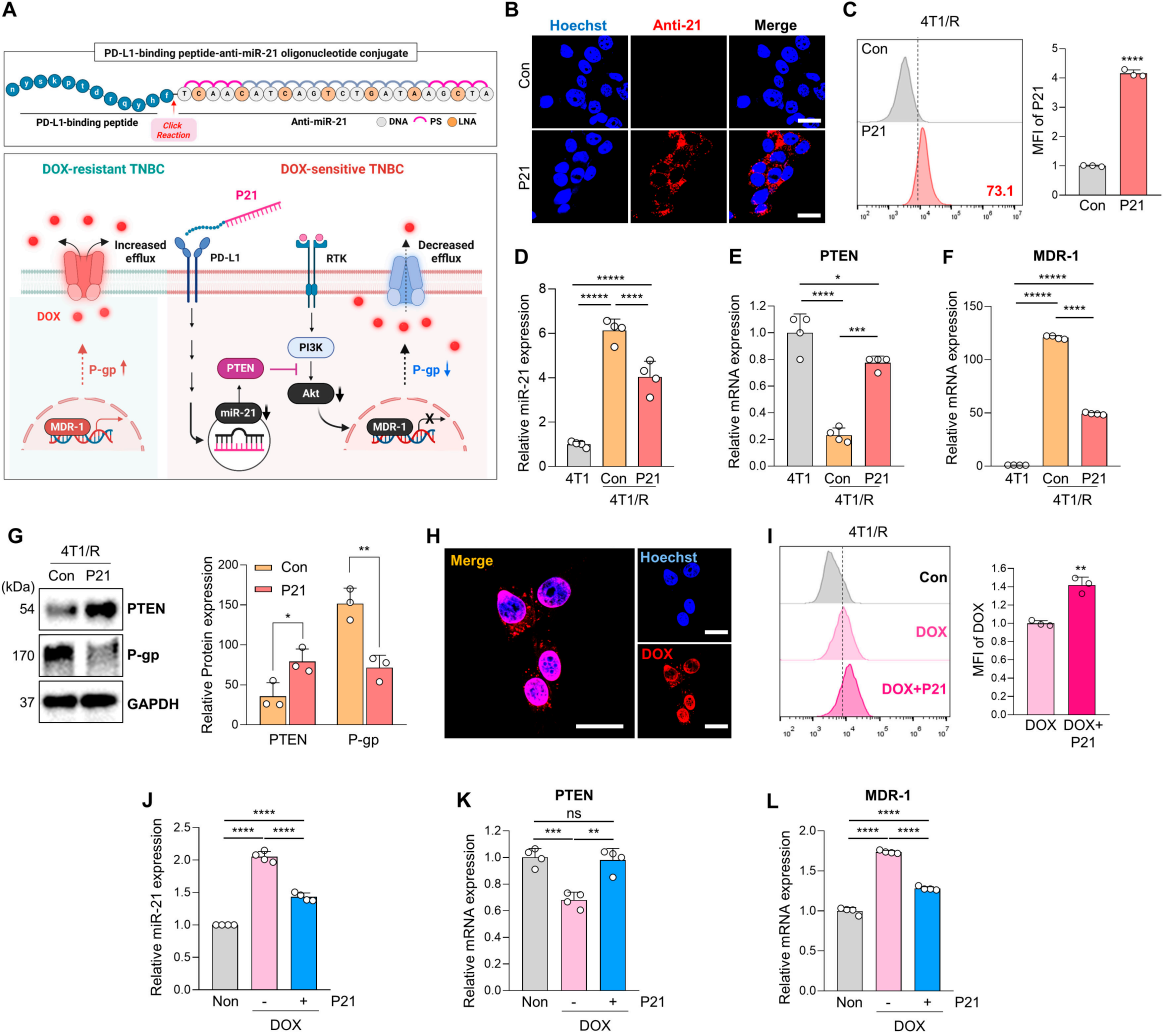
Overcoming DOX resistance by miR-21-related efflux pump deactivation. (A) Schematic illustration of overcoming DOX sensitivity by suppressing miR-21 levels in DOX-resistant TNBC cells. (B) Representative confocal images of uptake by 4T1/R cells after treatment with P21 (150 nM) for 6 h. The nuclei were stained with Hoechst 33342 (blue) (scale bar, 50 μm; *n* = 3/group). (C) Representative flow cytometric histogram of intracellular uptake of P21 in 4T1/R cells after treatment with P21 (150 nM) for 1 h. Data are presented as the relative MFI values (*n* = 3/group). (D) Relative miR-21 expression levels were measured by qRT-PCR in 4T1 and 4T1/R cells after treatment with P21 (150 nM) for 24 h. All samples were normalized to U6 expression (*n* = 4/group). (E and F) Relative PTEN and MDR-1 mRNA expression levels in 4T1 and 4T1/R cells after treatment with P21 (150 nM) for 24 h. All samples were normalized according to 18s RNA expression (*n* = 4/group). (G) Western blot analysis of PTEN and P-gp abundance in 4T1/R cells after treatment with P21 (150 nM) for 24 h (*n* = 3/group). (H) Representative confocal images of intracellular uptake of DOX by 4T1/R cells after preincubation with P21 (150 nM) for 24 h before treatment with DOX (2 μM) for 8 h. The nuclei were stained with Hoechst 33342 (blue) (scale bar, 50 μm; *n* = 3/group). (I) Representative flow cytometric histogram of intracellular uptake of DOX by untreated or P21-pretreated 4T1/R cells before treatment with DOX (2 μM) for 1 h. Data are presented as the relative MFI values (*n* = 3/group). (J) Relative miR-21 expression levels were measured by qRT-PCR in 4T1 cells after preincubation with P21 (150 nM) for 24 h, followed by a 24-h treatment with DOX (0.5 μM). All samples were normalized to U6 expression (*n* = 4/group). (K and L) Relative PTEN and MDR-1 mRNA expression levels in 4T1 cells after preincubation with P21 (150 nM) for 24 h, followed by a 24-h treatment with DOX (0.5 μM). All samples were normalized according to 18s RNA expression (*n* = 4/group).

PI3K/Akt signaling pathway has been linked to increased P-gp expression, resulting in the enhanced efflux of chemotherapeutic drugs from cancer cells and contributing to MDR [32,33]. To address this issue in cancer treatment, we aimed to deactivate P-gp activity by regulating the PTEN/phosphatidylinositol 3-kinase (PI3K)/Akt signaling pathway (Fig. 2A). Recognizing miR-21’s key role in DOX resistance, we hypothesized that targeted delivery of anti-miR-21 to DOX-resistant 4T1 cells could elevate intracellular DOX concentrations by down-regulating efflux pump activity, thereby improving chemotherapy efficacy. To test our hypothesis, we employed the P21 construct, previously established as a delivery platform for anti-miR-21 [30]. In brief, P21 was prepared by the conjugation of the azidoacetylated-modified PD-L1-binding peptide (N_3_-nyskptdrqyhf, d-form) with diarylcyclooctyne (DBCO)-functionalized anti-miR-21 (DBCO-5′-TCAACATCAGTCTGATAAGCTA-3′) via a cupper-free click reaction at a ratio of 2:1. Before confirming the therapeutic potential of P21 in DOX-resistant 4T1 cells, we assessed the cellular uptake of Cy5-labeled anti-miR-21 in 4T1/R cells. Due to PD-L1 overexpression on DOX-resistant cell surfaces, remarkable cellular uptake of P21 by 4T1/R cells was observed within 6 h (Fig. 2B). Consistent with confocal microscopy results, flow cytometry analysis also quantified excellent cellular uptake of P21 by 4T1/R cells (Fig. 2C).

Next, we evaluated whether P21 could effectively overcome DOX resistance by modulating the PTEN and P-gp signaling pathways. As shown in Fig. 2D, P21-treated 4T1/R cells showed significant inhibition of miR-21 levels compared to untreated cells. Moreover, after 24 h of P21 treatment in 4T1/R cells, there was a dramatic increase in PTEN expression and a decrease in P-gp expression observed at both mRNA and protein levels (Fig. 2E to G). Considering P-gp’s role in DOX efflux, lowering P-gp levels is expected to enhance DOX sensitivity in 4T1/R cells. Indeed, pretreating 4T1/R cells with P21 showed a notable increase in DOX accumulation within the 4T1/R cells (Fig. 2H). Flow cytometry results also confirmed a 1.3-fold increase in intracellular DOX accumulation in P21-pretreated 4T1/R cells compared to those treated with DOX alone (Fig. 2I). These findings underscore the importance of reducing miR-21 levels in DOX-resistant 4T1 cells to improve DOX sensitivity and increase intracellular DOX accumulation by deactivating P-gp activity. Finally, to explore the preventive effects of P21, we pretreated 4T1 cells with P21 before exposing them to DOX and analyzed changes in gene expression related to DOX resistance pathways. The results showed that P21 pretreatment maintained the expression levels of key genes, such as miR-21, PTEN, and MDR-1, at levels comparable to those in the nontreated control group (Non). Therefore, these findings suggest that P21 is effective in both preventing and overcoming DOX resistance (Fig. 2J to L).

### Inhibition of miR-21 enhances the cytotoxic effects of DOX on 4T1/R cells

**Fig. 3.**
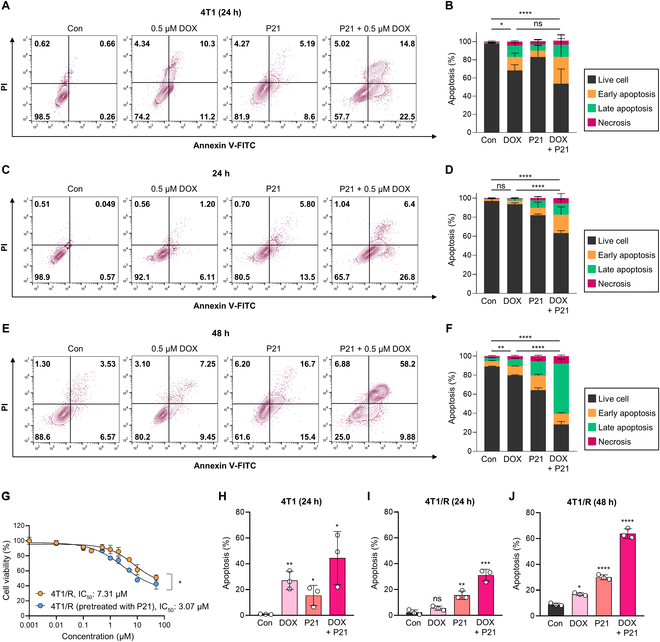
P21-mediated enhancement of DOX sensitivity in 4T1/R cells. (A and B) Representative flow cytometric data of Annexin V/PI in 4T1 cell lines after treatment with DOX (0.5 μM), P21 (150 nM), and P21 + DOX for 24 h (*n* = 3/group). (C and D) Representative flow cytometric data of Annexin V/PI in 4T1/R cell lines after treatment with DOX (0.5 μM), P21 (150 nM), and P21 + DOX for 24 h (*n* = 3/group). (E and F) Representative flow cytometric data of Annexin V/PI in 4T1/R cell lines after treatment with DOX (0.5 μM), P21 (150 nM), and P21 + DOX for 48 h (*n* = 3/group). (G) Viability of 4T1/R cells following treatment with gradient doses of DOX (0 to 100 μM) for 24 h after preincubation with P21 (150 nM) for 24 h. (H to J) Quantitative analysis of cell apoptosis as shown in (A) to (F).

To examine the P21-mediated enhancement of DOX sensitivity in 4T1/R cells, we measured apoptosis using Annexin V/PI staining. In DOX-sensitive 4T1 cells, treatment with 0.5 μM DOX for 24 h resulted in a 21.5% apoptosis rate and a corresponding viability rate of 74.2%, demonstrating that these cells are highly susceptible to DOX-induced apoptosis (Fig. 3A and B). In contrast, 4T1/R cells treated with the same concentration of DOX for 24 and 48 h showed minimal impact on the percentage of live cells (92.1% and 80.2%, respectively), with no marked increase in apoptosis compared to the PBS-treated control group (Con) (Fig. 3C to F). These results indicate that P-gp-mediated DOX efflux contributes to the reduced accumulation of DOX in 4T1/R cells, leading to resistance to DOX-induced cell apoptosis. Next, we investigated whether the reduction in miR-21 levels triggers DOX-induced apoptosis. While treatment with P21 alone induced mild cytotoxic impact on the cells after 48 h, P21 combined with DOX significantly enhanced apoptosis induction in 4T1/R cells, with rates of 33.2% and 68.08%, respectively, in the time-dependent manner. Consequently, these results demonstrate that the down-regulation of miR-21 improved the DOX sensitivity in 4T1/R cells by modulating the DOX efflux pump involved in DOX resistance. Additionally, we assessed the viability of 4T1/R cells treated with various concentrations of DOX for 24 h, following a 24-h preincubation with P21. As expected, P21 significantly enhanced DOX sensitivity in 4T1/R cells, reducing the IC_50_ value from 7.31 μM in untreated cells to 3.07 μM (Fig. 3G). Since P21 pretreatment restores DOX sensitivity in resistant cell lines, administering DOX alone to 4T1/R cells for 24 h did not result in a statistically significant increase in cell apoptosis compared to 4T1 cells (Fig. 3H and I). Although prolonged exposure to DOX for 48 h in 4T1/R cells did induce some apoptosis, it was minimal compared to the substantial apoptosis observed in DOX-sensitive 4T1 cells (Fig. 3J). In contrast, the combination of P21 with DOX enhanced cell apoptosis, quantitatively confirming that P21 effectively reverses DOX resistance.

### P21 reverses P-gp-mediated DOX resistance by reactivating PTEN level in a murine 4T1 xenograft model

**Fig. 4.**
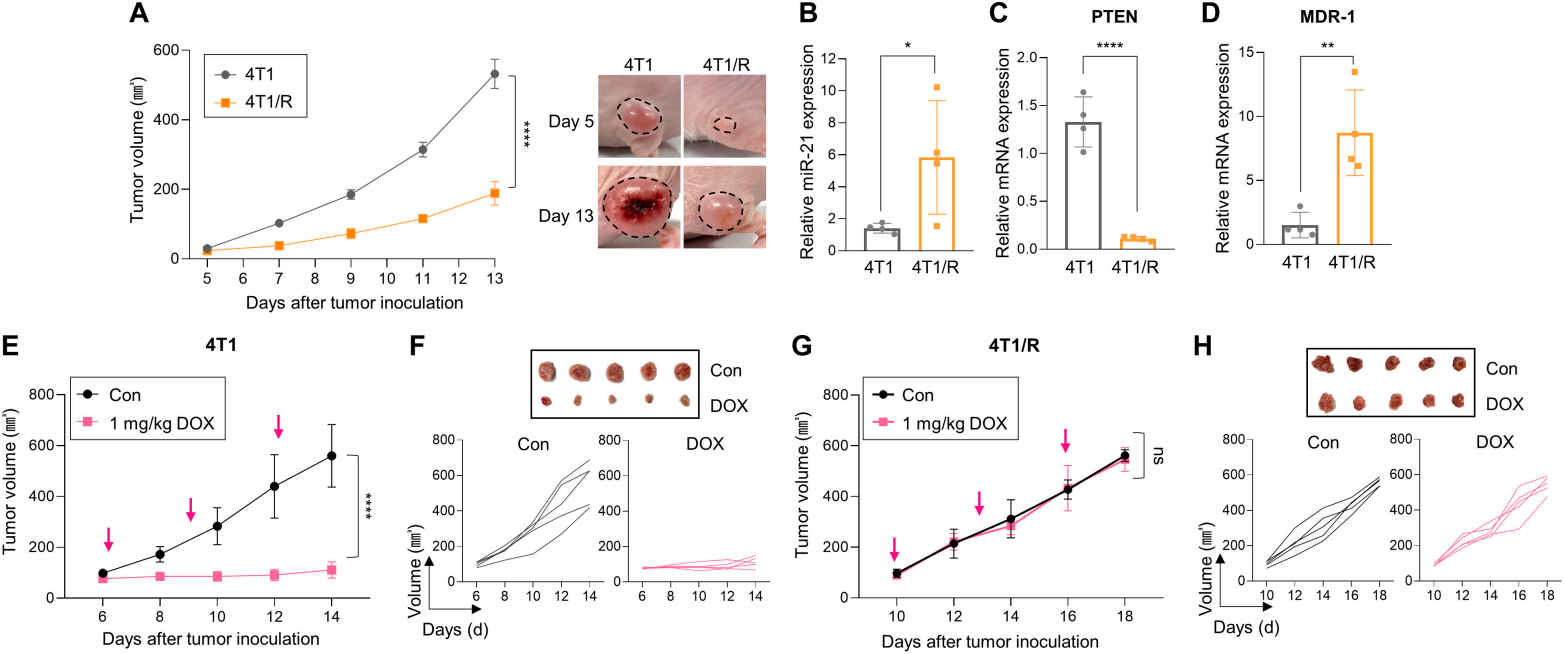
Establishment of a DOX-resistant murine 4T1 xenograft model. (A) Tumor growth in 4T1 and 4T1/R tumor-bearing mice models. (B) Relative miR-21 expression levels were measured by qRT-PCR in tumor tissues. All samples were normalized to U6 expression (*n* = 4/group). (C and D) Relative PTEN and MDR-1 mRNA expression levels in tumor tissues. All samples were normalized according to 18s RNA expression (*n* = 4/group). (E) Tumor growth in 4T1 tumor-bearing mice over 14 d after tumor inoculation (*n* = 5/group). (F) Representative tumor images collected from 4T1 tumor-bearing mice on day 14 after treatment with DOX (1 mg/kg). (G) Tumor growth in 4T1/R tumor-bearing mice over 18 d after tumor inoculation (*n* = 5/group). (H) Representative tumor images collected from 4T1/R tumor-bearing mice on day 18 after treatment with DOX (1 mg/kg).

Prior to verifying the in vivo anticancer efficacy of P21, we established murine models harboring 4T1/R tumors, which consistently exhibited resistance to DOX. Upon monitoring tumor growth rates following transplantation of each 4T1 and 4T1/R cell into the flank of nude mice, we observed delayed tumor growth in 4T1/R tumors compared to the 4T1 tumor-bearing mouse model (Fig. 4A). Tumor tissues were collected from both mouse models on day 13 after tumor inoculation to explore their DOX-resistant characteristics. Corresponding to in vitro findings, miR-21 expression was significantly higher in the 4T1/R tumor tissues compared to the 4T1 tumor tissues (Fig. 4B). Additionally, a marked decrease in PTEN levels and a notable increase in MDR-1 levels at the mRNA level were observed in the 4T1/R tumor-bearing mouse model (Fig. 4C and D). Subsequently, we evaluated the sensitivity of both cell lines to DOX. In the 4T1 tumor-bearing model, DOX treatment effectively suppressed tumor growth (Fig. 4E and F). However, in the 4T1/R tumor-bearing mouse model, DOX treatment failed to inhibit tumor growth due to impaired sensitivity of the tumor to DOX (Fig. 4G and H).

**Fig. 5.**
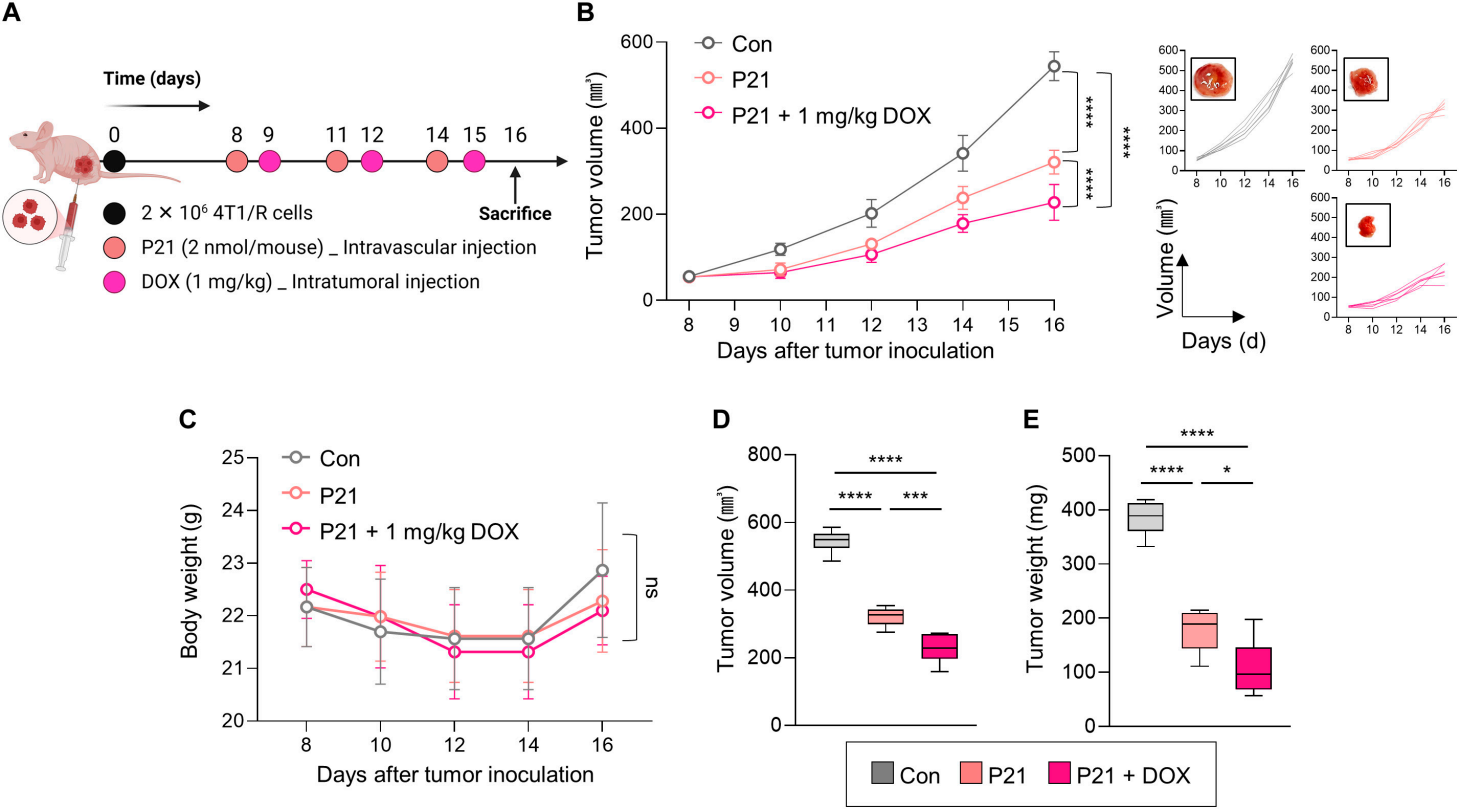
In vivo therapeutic efficacy of combined therapy with P21 and DOX in a DOX-resistant murine 4T1 xenograft model. (A) Schematic of the combination therapy of P21 (0.78 mg/kg) and DOX (1 mg/kg) in 4T1/R tumor-bearing mice models. (B) Tumor growth in 4T1/R tumor-bearing mice over 16 d after tumor inoculation (*n* = 6/group). (C) Body weight changes of mice for each group (*n* = 6/group). (D) Tumor volume (mm^3^) and (E) tumor weight at day 16 were analyzed (*n* = 6/group).

In light of the aforementioned findings, the importance of targeting miR-21 as a potential therapeutic strategy is underscored by its ability to overcome DOX resistance through the regulation of the P-gp efflux pump. Hence, we evaluated whether P21 could enhance intracellular DOX accumulation and ultimately restore sensitivity to DOX in a DOX-resistant murine 4T1 xenograft model. After the tumor volume reached approximately 50 to 60 mm^3^, mice bearing 4T1/R tumors were administered 0.78 mg/kg P21 via intravenous injection and 1 mg/kg DOX via intratumoral injection, respectively (Fig. 5A). Surprisingly, the combined treatment of P21 and DOX exerted a more dramatic tumor inhibition effect without significant body weight change (Fig. 5B and C). Compared to the P21 monotherapy group, the combination of DOX with P21 exhibited remarkable reductions in both the extracted tumor volume and tumor weight, with changes of 0.29-fold and 0.39-fold, respectively (Fig. 5D and E). The combination therapy of P21 and DOX synergistically suppressed tumor growth, indicating that P21’s dual action, not only independently inhibiting tumor growth but also restoring sensitivity to DOX, contributed to this effect [30].

**Fig. 6.**
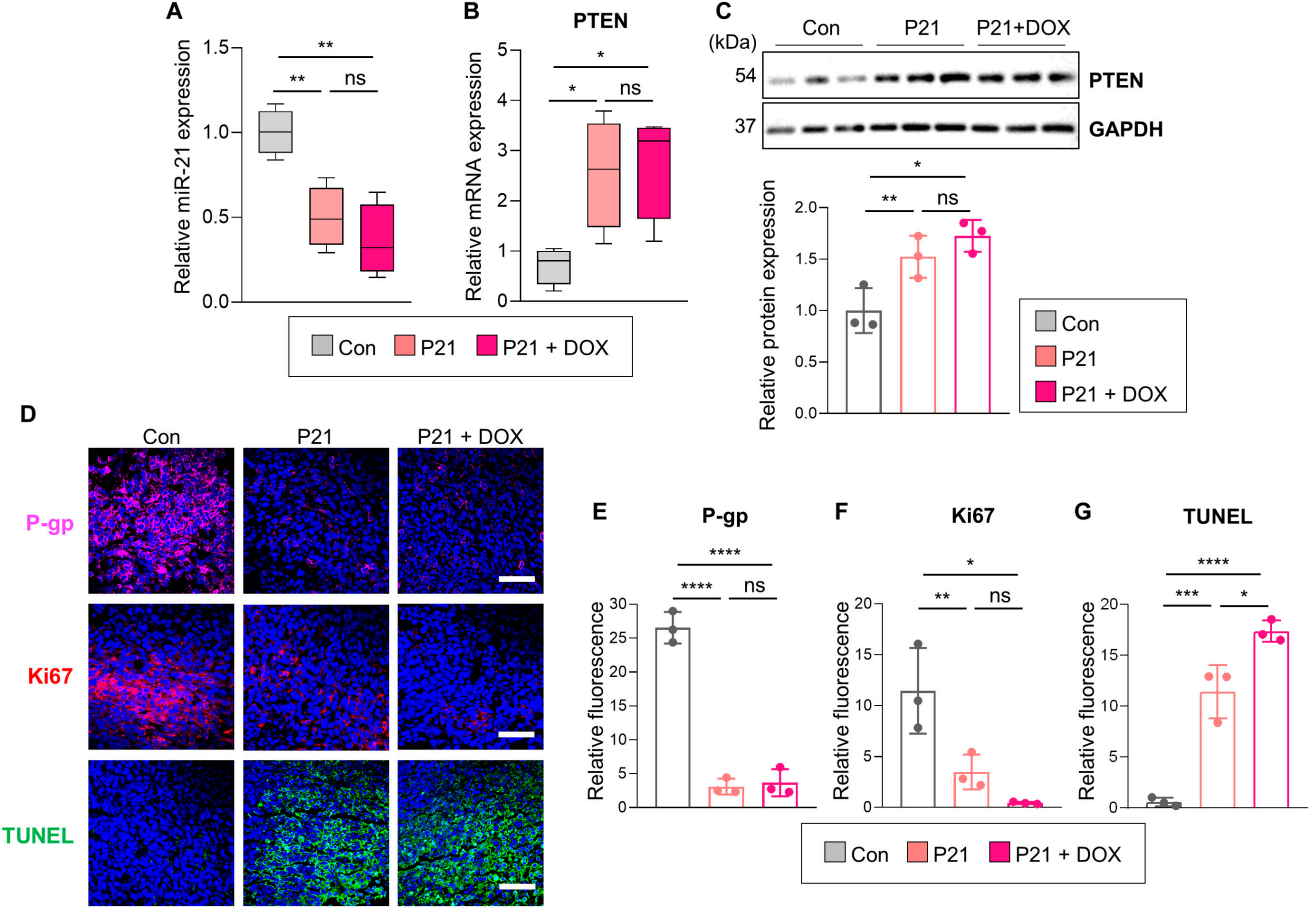
Overcoming P-gp-mediated DOX resistance by P21 treatment in a DOX-resistant murine 4T1 xenograft model. (A) Relative miR-21 expression levels were measured by qRT-PCR in tumor tissues. All samples were normalized to U6 expression (*n* = 4/group). (B) Relative PTEN mRNA expression level in tumor tissues. All samples were normalized according to 18s RNA expression (*n* = 4/group). (C) Western blot analysis of PTEN abundance in tumor tissues (*n* = 3/group). (D) Representative immunofluorescence images of P-gp (pink), Ki67 (red) expression, and TUNEL (green) in tumor tissues (scale bar, 50 μm; *n* = 3/group). The nuclei were stained with Hoechst 33342 (blue). (E to G) Quantification of P-gp, Ki67, and TUNEL fluorescence intensity.

Furthermore, we found that P21 markedly down-regulated miR-21 expression compared to the PBS-treated control group, triggering the reactivation of PTEN levels at both the mRNA and protein levels (Fig. 6A to C). Histological analyses also revealed a notable reduction in P-gp levels in the P21-treated groups, indicating that P21 effectively attenuated drug efflux capability and restored DOX sensitivity in a DOX-resistant 4T1 model (Fig. 6D and E). Immunofluorescence staining for Ki67 and TUNEL was also conducted to verify whether P21 effectively promotes DOX-mediated apoptosis. Ki67 expression, as a marker of cancer cell proliferation, was notably lower in the P21-treated groups compared to the PBS-treated control group (Fig. 6F). Additionally, a substantial increase in the proportion of TUNEL-positive cells was observed, indicating heightened levels of apoptosis induced by DOX (Fig. 6G). These findings underscore the significance of P21 in inhibiting cancer cell proliferation and restoring sensitivity to DOX, surpassing its anticipated role in tumor suppression. Collectively, our findings validate that P21 effectively overcomes P-gp-mediated DOX resistance by reactivating PTEN levels in a DOX-resistant murine 4T1 xenograft model.

## Discussion

Chemotherapy has significantly improved survival rates in TNBC patients; however, the emergence of MDR poses a significant challenge, leading to treatment failures. While aberrant miRNA expression is frequently observed in TNBC, understanding their precise roles in chemoresistance remains incomplete due to the complex signaling pathways.

Among these miRNAs, miR-21 stands out as a key player in promoting drug resistance through various mechanisms. Research reveals that miR-21 inhibits apoptosis, stimulates cell proliferation, and modulates critical signaling pathways like PI3K/Akt, mitogen-activated protein kinase (MAPK), and transforming growth factor-β (TGF-β), all vital in drug sensitivity [26,34]. Furthermore, miR-21 orchestrates the expression of genes associated with drug resistance, including anti-apoptotic proteins, DNA repair enzymes, and drug-metabolizing enzymes, reshaping cellular response to chemotherapy [35]. Despite efforts, challenges persist in delivering miR-21 inhibitors due to a lack of target specificity, similar to earlier generations of P-gp inhibitors. Hence, understanding miR-21’s role as a biomarker and therapeutic target is crucial for the efficient delivery of anti-miR-21 to TNBC cells through precise targeting modalities.

Notably, miR-21 also affects genes involved in drug transport and metabolism, reducing intracellular drug accumulation and contributing to resistance. Our study elucidates miR-21’s P-gp inhibitory mechanism in TNBC, revealing its role in reducing intracellular DOX accumulation and enhancing chemoresistance. Tumor-specific knockdown of miR-21 by P21 significantly mitigates DOX resistance in TNBC cells by targeting the MDR-1 mRNA through PTEN regulation. Our previous studies have demonstrated that anti-miR-21 treatment effectively lowers miR-21 levels over the long term, without significant off-target effects or toxicity, highlighting its potential as an effective cancer therapy [36,37]. However, detailed studies on the long-term effects and safety of anti-miR-21 treatment are crucial for its clinical application. These findings suggest miR-21 as a pivotal regulator of drug resistance in breast cancer, offering insights into novel therapeutic strategies for overcoming chemotherapy resistance and improving treatment outcomes in TNBC patients. While our experiments specifically focused on the 4T1 TNBC model, previous studies suggest that reactivating PTEN is crucial for overcoming drug resistance in TNBC. PTEN plays a key role in modulating the PI3K/Akt/mammalian target of rapamycin (mTOR) signaling pathway, which is often dysregulated in other TNBC models [27,28]. As shown in other studies, miR-21 is markedly up-regulated in drug-resistant TNBC models, such as MDA-MB-231 cell lines, revealing its role as a crucial therapeutic target in the regulation of drug resistance [29]. Therefore, these findings indicate that the therapeutic effects of targeting miR-21 could extend beyond the 4T1 model, potentially offering an effective strategy for overcoming drug resistance across various TNBC models.

## Data Availability

Data will be made available on request.
